# Cost-effectiveness analysis of adebrelimab combined with chemotherapy for extensive-stage small cell lung cancer

**DOI:** 10.3389/fphar.2022.1019826

**Published:** 2022-10-26

**Authors:** Maojin You, Ruijia Chen, Qingfeng Wu, Wei Zhu, Ying He, Yufan Huang

**Affiliations:** ^1^ Department of Pharmacy, Mindong Hospital Affiliated to Fujian Medical University, Ningde, China; ^2^ Department of Pharmacy, Mengchao Hepatobiliary Hospital of Fujian Medical University, Fuzhou, China; ^3^ Department of Emergency Medicine, Mindong Hospital Affiliated to Fujian Medical University, Ningde, China

**Keywords:** cost-effectiveness, adebrelimab plus chemotherapy, extensive-stage small cell lung cancer, first-line treatment, small cell lung cancer

## Abstract

**Background:** The findings of the CAPSTONE-1 trial showed that adebrelimab in combination with chemotherapy (etoposide-carboplatin) (ADCHM) is clinically beneficial as a first-line treatment for patients with extensive-stage small cell lung cancer (ES-SCLC), compared with placebo plus chemotherapy (PLCHM, etoposide-carboplatin). However, owing to the higher cost of adebrelimab, it is unclear whether ADCHM is cost-effective compared with PLCHM. This study aimed to evaluate the cost-effectiveness of ADCHM as a first-line treatment for patients with ES-SCLC from the perspective of the Chinese healthcare system.

**Methods:** A Markov model with three health states was developed to assess the cost-effectiveness of ADCHM as a first-line treatment option with ES-SCLC. Clinical data were obtained from the CAPSTONE-1 trial. Costs of the drug were calculated at national tender prices, and other costs and utility values were obtained from published literature. The outcomes included life years (LYs), quality-adjusted life years (QALYs), and incremental cost-effectiveness ratios (ICERs). One-way sensitivity analysis and probabilistic sensitivity analysis were used to validate the robustness of the model.

**Results:** The ADCHM group achieved 1.21 QALYs (2.47 LYs) for $25,312, whereas the PLCHM group achieved 0.81 QALYs (1.59 LYs) for $14,846. The ICER for ADCHM *versus* PLCHM was $25914 per QALY gained. The variables with the greatest impact on the model results were the utility value of progressive disease, the utility value of progression-free survival, and the price of adebrelimab (100 mg). At a willingness-to-pay threshold of $37,653/QALY, ADCHM had an 89.1% probability of being cost-effective compared with PLCHM.

**Conclusion:** ADCHM may be a cost-effective first-line treatment strategy for ES-SCLC from the perspective of the Chinese healthcare system.

## 1 Introduction

Worldwide, lung cancer has the second most frequent incidence and is the leading cause of cancer-related mortality, with approximately 1.8 million deaths reported in 2020, i.e., approximately 20% of all cancer deaths (Sung et al., 2021). Small cell lung cancer (SCLC), the most lethal subtype of lung cancer (Oronsky et al., 2017), has a 5-year survival rate of less than 7% (Karachaliou et al., 2016) and accounts for approximately 15% of all lung cancer types (Rudin et al., 2021); nearly two-thirds of SCLC cases progress to the extensive stage at the initial diagnosis (Oronsky et al., 2017). The median overall survival (OS) of patients with untreated extensive-stage SCLC (ES-SCLC) is dismal, at 2–4 months (Liu et al., 2020). Platinum-based drugs combined with etoposide chemotherapy are the standard treatment for ES-SCLC, however, the median OS is merely 9–11 months (Liu et al., 2020). Therefore, developing new treatment regimens for ES-SCLC is an urgent task.

Immune checkpoint inhibitors (ICIs) reduce immunosuppression in the tumor microenvironment and reactivate the anti-tumor function of T cells by inhibiting cytotoxic T lymphocyte-associated protein 4 and programmed cell death-1 pathway/programmed cell death receptor ligand-1 (PD-L1) (Kang et al., 2021). ICIs yield effective results in ES-SCLC treatment (Horn et al., 2018; Paz-Ares et al., 2019; Rudin et al., 2020), bringing new hope for survival among patients with ES-SCLC.

Adebrelimab, an ICI developed in China, is a human anti-PD-L1 monoclonal antibody. Wang et al. (2022) conducted a phase III clinical trial (CAPSTONE-1) in China to estimate the efficacy and safety of adebrelimab combined with chemotherapy (ADCHM) *versus* placebo combined with chemotherapy (PLCHM, carboplatin-etoposide) as the first-line treatment for ES-SCLC. The outcomes showed that as compared to PLCHM, ADCHM significantly improved the OS in previously untreated patients with ES-SCLC.

Although ADCHM offers clinical benefits for patients with ES-SCLC, its high cost limits its widespread use. Therefore, it is essential to evaluate the cost-effectiveness of ADCHM through a pharmacoeconomic approach to estimate the clinical benefits and potential financial consequences of ADCHM for patients with ES-SCLC and determine the rationale for its widespread use in the future. To our knowledge, no economic evaluations of ADCHM treatment for ES-SCLC have been conducted. Our study assessed the cost-effectiveness of ADCHM as a first-line treatment option for ES-SCLC from the perspective of the Chinese healthcare system based on the published results of the CAPSTONE-1 trial (Wang et al., 2022).

## 2 Materials and methods

### 2.1 Model construction

The study was designed following the Consolidated Health Economic Evaluation Reporting Standards (CHEERS) reporting guidelines (Supplementary Table SA) (Husereau et al., 2022). The probabilities of progression-free survival (PFS) and OS were extracted using corresponding Kaplan-Meier survival curves from two treatment groups (ADCHM and PLCHM groups) in the CAPSTONE-1 trial by GetData Graph Digitizer (version 2.26) (Wan et al., 2019; Wang et al., 2022). Statistical analyses were performed using the R software (version 4.2.0) packages, “survival”, “survHE”, and “survminer.” Individual patient data were reconstructed into each Kaplan-Meier curve, and the data were fitted by the survival analysis method described by Hoyle et al. (Hoyle and Henley, 2011). The observation period and subsequent survival functions were obtained by fitting and extrapolating the Kaplan-Meier curves. The distribution functions (including exponential, Weibull, log-normal, and log-logistic) were examined to select the best-fit survival functions using the Akaike information criterion (AIC) and Bayesian information criterion (BIC), i.e., lower AIC and BIC values indicated a better fit (Ishak et al., 2013; Williams et al., 2017), and these values for various survival distribution functions for the PFS and OS curves are shown in Supplementary Table SB. Ultimately, the log-logistic distribution function, (S(t)= (1+(λt)^γ^)^−1^; S: survival probability, t: time cycle, λ: scale parameter, and γ: shape parameter), provided the best fit for PFS and OS data and was used to generate corresponding transition probabilities for ADCHM and PLCHM strategies (Table 1).

**TABLE 1:** The basic parameters of the input model and the range of sensitivity analyses.

Variable	Base value	Range		Distribution	Source
		Min	Max		
Log-logistic survival model of PFS					
ADCHM group					
Scale (λ)	0.1489507	0.119161	0.178741	Log-logistic	Wang et al. (2022)
Shape (γ)	2.070122	1.656098	2.484146	Log-logistic	Wang et al. (2022)
PLCHM group					
Scale (λ)	0.1767604	0.141408	0.212112	Log-logistic	Wang et al. (2022)
Shape (γ)	3.377706	2.702165	4.053247	Log-logistic	Wang et al. (2022)
Log-logistic survival model of OS					
ADCHM group					
Scale (λ)	0.06284631	0.050277	0.075416	Log-logistic	Wang et al. (2022)
Shape (γ)	1.924522	1.539618	2.309426	Log-logistic	Wang et al. (2022)
PLCHM group					
Scale (λ)	0.07650712	0.061206	0.091809	Log-logistic	Wang et al. (2022)
Shape (γ)	2.665497	2.132398	3.198596	Log-logistic	Wang et al. (2022)
ADCHM: Incidence of AEs					
Neutrophil count decreased	0.757	0.606	0.908	Beta	Wang et al. (2022)
White blood cell count decreased	0.461	0.369	0.553	Beta	Wang et al. (2022)
Platelet count decreased	0.383	0.306	0.460	Beta	Wang et al. (2022)
Anemia	0.278	0.222	0.334	Beta	Wang et al. (2022)
PLCHM: Incidence of AEs					
Neutrophil count decreased	0.754	0.603	0.905	Beta	Wang et al. (2022)
White blood cell count decreased	0.379	0.303	0.455	Beta	Wang et al. (2022)
Platelet count decreased	0.336	0.269	0.403	Beta	Wang et al. (2022)
Anemia	0.284	0.227	0.341	Beta	Wang et al. (2022)
Cost ($)					
Neutrophil count decreased	84.21	67.37	101.05	Gamma	Li et al. (2021)
White blood cell count decreased	466.00	372.80	559.20	Gamma	Zhang et al. (2021)
Platelet count decreased	1054.00	843.20	1264.80	Gamma	Peng et al. (2022)
Anemia	508.20	406.56	609.84	Gamma	Zhang et al. (2021)
Carboplatin (100 mg)	4.10	3.28	4.92	Gamma	(Yaozhi Net, 2022)
Etoposide (100 mg)	1.21	0.97	1.45	Gamma	(Yaozhi Net, 2022)
Irinotecan (100 mg)	274.90	219.92	329.88	Gamma	(Yaozhi Net, 2022)
Cisplatin (100 mg)	11.74	9.39	14.09	Gamma	(Yaozhi Net, 2022)
Adebrelimab (100 mg)	25.77	20.62	30.92	Gamma	Yaozhi Net, (2022)
Routine follow-up per cycle	73.86	59.09	88.64	Gamma	Kang et al. (2021)
Tests per cycle	152.09	121.67	182.51	Gamma	Kang et al. (2021)
Best supportive care per cycle	359.00	287.20	430.80	Gamma	Kang et al. (2021)
End-of-life care	2176.00	1740.80	2611.20	Gamma	Kang et al. (2021)
Utility value					
PFS	0.673	0.538	0.808	Beta	Kang et al. (2021)
PD	0.473	0.378	0.568	Beta	Kang et al. (2021)
Disutility due to AEs					
Neutrophil count decreased	0.20	0.16	0.24	Beta	Nafees et al. (2017)
White blood cell count decreased	0.20	0.16	0.24	Beta	Nafees et al. (2017)
Platelet count decreased	0.19	0.15	0.23	Beta	Nafees et al. (2017)
Anemia	0.073	0.058	0.088	Beta	Nafees et al. (2017)
Body surface area (m^2^)	1.72	1.38	2.06	Normal	Zhang P. F et al. (2020)
Creatinine clearance rate (ml/min)	70	52.5	87.5	Gamma	Liu et al. (2021b)
Discount rate (%)	5	0	8	Fixed	Liu et al. (2011)
Proportion					
Receiving chemotherapy in the ADCHM group	0.40	0.32	0.48	beta	Wang et al. (2022)
Receiving chemotherapy in the PLCHM group	0.52	0.42	0.62	beta	Wang et al. (2022)
Receiving best supportive care in the ADCHM group	0.60	0.48	0.72	beta	Wang et al. (2022)
Receiving best supportive care in the PLCHM group	0.48	0.38	0.58	beta	Wang et al. (2022)

#The price of Adebrelimab is assumed based on the price of sintilimab; ADCHM: adebrelimab in combination with chemotherapy (etoposide-carboplatin); AE, adverse event; PD: progressive disease; PFS: Progression-free survival; PLCHM: placebo plus chemotherapy (etoposide-carboplatin).

**FIGURE 1:**
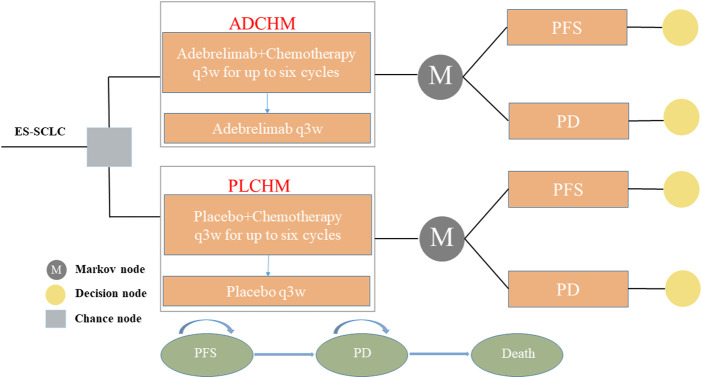
The Markov model simulating outcomes for the CAPSTONE-1 trial. All patients with ES-SCLC started with PFS state and received treatment with ADCHM or PLCHM. ADCHM: adebrelimab in combination with chemotherapy (etoposide-carboplatin); ES-SCLC: extensive-stage small-cell lung cancer. PD: progressive disease; PFS: Progression-free survival; PLCHM: placebo plus chemotherapy (etoposide-carboplatin).

To simulate the cost and effectiveness of ADCHM as a first-line treatment for ES-SCLC compared with PLCHM, a Markov model was developed using TreeAge Pro 2022 (TreeAge Software, Williams-town, MA, United States). The model included three mutually exclusive health states, namely PFS, progressive disease (PD), and death (Figure 1). The time horizon of the model was 6.9 years (approximately 120 cycles), which was determined by the expected time for 99% of the hypothetical patients modeled to die. The cycle length was 21 days. During each cycle, patients either maintained their assigned health state or progressed to a new health state and were not allowed to return to their previous healthy state. The background mortality in China was not considered in our model, owing to the high lethality of ES-SCLC. According to the method described by Li et al. (2022), the transition probability from the PFS state to the death state was assumed as 0 in the Markov model, i.e., there was no direct transition from the PFS state to the death state. Total costs, life years (LYs), quality-adjusted life years (QALYs), and incremental cost-effectiveness ratios (ICERs) were the output data obtained from our model. Our cost-effectiveness analysis was conducted from the perspective of the Chinese healthcare system. We set the willingness-to-pay (WTP) threshold at $37,653/QALY (three times the gross domestic product per capita in China in 2021), as recommended by the World Health Organization, and the treatment regimen was considered cost-effective if the ICER was below our predefined WTP threshold.

### 2.2 Clinical data

Clinical data were extracted from CAPSTONE-1, a phase III randomized controlled clinical trial conducted across 47 tertiary hospitals in China. Patients were enrolled based on the following criteria: 1) 18–75-year-old individuals with histologically or cytologically confirmed ES-SCLC; 2) those who were not treated previously with systemic therapy; 3) Eastern Cooperative Oncology Group (ECOG) performance status score of 0 or 1; 4) Response Evaluation Criteria in Solid Tumors (version 1.1) based inclusion, and at least 3 months of life expectancy (Supplementary Figure SB) (Wang et al., 2022). These patients randomly received ADCHM or PLCHM regimens. Carboplatin (area under the curve of 5 mg/mL/min) and etoposide (100 mg/m^2^ of body surface area) were administered per cycle for up to six cycles. Parallelly, adebrelimab (20 mg/kg) and placebo were administered to patients in the ADCHM group and the PLCHM group, respectively, until disease progression or unacceptable toxicity, for up to 24 months. Treatment with adebrelimab was discontinued in 5.2% of patients in the ADCHM group due to treatment-related adverse events for a median treatment duration of 8 cycles according to the CAPSTONE-1 trial (Wang et al., 2022). Further, to simplify the model, after patients developed disease progression, it was assumed that some patients received second-line chemotherapy (irinotecan + cisplatin), while the remaining received the best supportive care (Table 1). The CAPSTONE-1 trial (Wang et al., 2022) does not report the implementation of second-line chemotherapy, and thus, we utilized the results of Zhao et al. (2019), who conducted a retrospective study for assessing the efficacy of second-line chemotherapy in SCLC patients whereby the first-line standard therapy failed, to estimate the duration of chemotherapy (approximately 3.6 cycles) required for these patients. Each patient received the best supportive care after the failure of second-line therapy. In the CAPSTONE-1 trial, the median age of the patients was 62 years; therefore, we assumed a body surface area of 1.72 m^2^ (weight, 65 kg; height, 1.64 m) and a creatinine clearance rate of 70 ml/min to set the administration dose (Goulart and Ramsey, 2011; Zhu et al., 2018; Wang et al., 2022).

### 2.3 Costs and utilities

We only considered the direct medical costs, including the cost of drugs, tests, follow-up, end-of-life care, and management of adverse reactions of grade 3 or higher with an incidence greater than 5% (Table 1). The cost of drugs was obtained from the national tender prices (Yaozhi Net, 2022). However, adebrelimab is not yet on the market, and thus, we could not obtain its exact price. We estimated the plausible price of adebrelimab in China (converted to the price required per cycle) based on the price of sintilimab (Yaozhi Net, 2022), a drug developed in China ($334.9/200 mg). Other costs were sourced from published literature and adjusted to the prices in 2021 using the China Statistics Bureau Medical Price Index (National Bureau of Statistics, 2021). All costs were converted using the average exchange rate in 2021 and expressed in US dollars ($1 = 6.45 RMB). It should be pointed out that apart from body weight, body surface area, and creatinine clearance, no other parameters can affect the cost of drugs. As the relevant data on the quality of life were not available in the CAPSTONE-1 trial (Wang et al., 2022), the utility of PFS and PD was assessed from published literature in China (Table 1) (Kang et al., 2021). We considered the disutility of adverse reactions of grade 3 or higher with an incidence greater than 5% to reduce the impact of using the same utility values for both treatment groups in the model. Both costs and health utilities were discounted, and the discounted values were set at 5% per year (Table 1) (Liu et al., 2011).

### 2.4 Sensitivity analysis

To examine the robustness of the model, we conducted sensitivity analyses, including one-way and probabilistic sensitivity analysis (PSA). We performed a one-way sensitivity analysis for each variable to determine the factors that directly affected the ICER and the final results were presented in a tornado diagram. We adjusted the variables within a given range (Table 1). The range of all variables was their 95% CIs derived from the literature or assumed to be ± 20% of the baseline value in cases of lack of data. The lower and upper bounds of the discount rate were set at 0% and 8%, respectively (Liu et al., 2011). In PSA, to verify the effects of the parameters on the uncertainty of the results, 1,000 iterations were performed in the Monte Carlo simulations with all parameters assigned to appropriate distributions in the model. All probability and health utility parameters were assigned the beta distribution. The costs and creatinine clearance rates were assigned the gamma distribution. The body surface area was assigned the normal distribution. The relevant parameters in the distribution of PSA were calculated based on the baseline values and ranges of variation for the parameters (Table 1). Simultaneously, we repeated the calculation of the acceptable probabilities of cost-effectiveness with ADCHM by continuously increasing the price of adebrelimab. When the acceptable probability was less than 50%, at that point ADCEHM was no longer considered cost-effective as the first-line treatment for ES-SCLC as compared to chemotherapy. We used the prices of available imported ICIs, including pembrolizumab ($2777.98/100 mg) and nivolumab ($1434.11/100 mg) (Yaozhi Net, 2022), as the reference for adebrelimab to calculate the acceptable probabilities for ADCHM (converted by the dose required for one cycle). The results of PSA are represented as scatter plots and cost-effectiveness acceptability curves.

### 2.5 Subgroup analysis

Subgroup analyses were performed to assess the uncertainty in outcomes owing to different patient characteristics, including sex, age, ECOG performance status, smoking history, lactate dehydrogenase concentration at enrolment, liver metastases, brain metastases, disease stage, and PD-L1 tumor proportion score (Table 2). Due to the lack of sufficient survival data for each subgroup, for subgroup survival extrapolation, we assumed that all subgroups in the PLCHM group had the same survival function (log-logistic survival model) for PFS and OS and estimated the PFS and OS survival function for each subgroup of the ADCHM group based on the subgroup-specific hazard ratios (Table 2) extracted from the results of the CAPSTONE-1 trial (Wang et al., 2022), according to the method described by Hoyle et al. (2010). The ICERs and probabilities of cost-effectiveness acceptability were calculated for each subgroup. In the subgroup analysis, we did not change other parameters except for the subgroup-specific hazard ratios.

**TABLE 2:** Results of subgroup analyses.

Subgroup	PFS HR (95% CI)	OS HR (95% CI)	ICER ($/QALY)	Cost-effectiveness probability (%)
Sex				
Male	0.72 (0.57–0.90)	0.72 (0.57–0.92)	30357	73.7
Female	0.55 (0.33–0.90)	0.62 (0.37–1.05)	24584	87.9
Age				
<65 years	0.70 (0.54–0.91)	0.71 (0.54–0.93)	29505	77.1
≥65 years	0.62 (0.43–0.89)	0.70 (0.48–1.00)	26978	82.1
ECOG performance status				
0	0.62 (0.35–1.10)	0.83 (0.46–1.52)	30188	67.1
1	0.69 (0.56–0.87)	0.69 (0.55–0.87)	28657	78.1
Smoking history				
Current or former smoker	0.76 (0.60–0.96)	0.75 (0.59–0.95)	32887	63.4
Never smoked	0.44 (0.27–0.71)	0.59 (0.37–0.95)	21697	93.7
LDH concentration at enrolment				
≤ULN	0.70 (0.52–0.95)	0.59 (0.42–0.82)	27243	81.8
>ULN	0.64 (0.48–0.85)	0.83 (0.62–1.11)	29593	74.4
Liver metastases				
Yes	0.74 (0·51–1.07)	0.92 (0.65–1.31)	41617	46.3
No	0.64 (0.50–0.83)	0.61 (0.46–0.81)	26167	86.5
Brain metastases				
No	0.65 (0.53–0.81)	0.68 (0.55–0.85)	27283	85.1
Disease stage				
IV	0.68 (0.55–0.83)	0.72 (0.58–0.90)	28909	80.6
PD-L1 tumour proportion score				
<1%	0.68 (0.54–0.85)	0.66 (0.52–0.83)	27822	80.2
≥1%	0.70 (0.34–1.45)	0.72 (0.33–1.59)	33099	63.1

ECOG, eastern cooperative oncology group; HR, hazard ratio; LDH, lactate dehydrogenase; OS, overall survival; PD-L1, programmed cell death receptor ligand-1; PFS, progression-free survival; QALY, quality-adjusted life years; ULN, upper normal limit.

## 3 Results

### 3.1 Base case analysis

The results of our study are expressed as LYs, QALYs, and ICER. The ADCHM group achieved 2.47 LYs and 1.21 QALYs at $25,312. In the PLCHM group, the effectiveness was 1.59 LYs and 0.81 QALYs at $14,846. The average incremental effectiveness and cost in the ADCHM group were 0.40 QALYs, and $10,466 respectively, relative to those in the PLCHM group. The ICER for ADCHM *versus* PLCHM was $25,914 per QALY gained (Table 3). At the WTP threshold of $37,653/QALY in China, ADCHM emerged as a more cost-effective treatment strategy than PLCHM.

**TABLE 3:** Effectiveness and costs obtained from the model.

Regimen	PLCHM	ADCHM	Incremental
Total cost, $	14,846	25,312	10,466
Overall LYs	1.59	2.47	0.88
Total QALYs	0.81	1.21	0.40
ICER, $			
Per LY			11,851
Per QALY			25,914

ADCHM: adebrelimab in combination with chemotherapy (etoposide-carboplatin); ICER: incremental cost-effectiveness ratio; LY: life year; PLCHM: placebo plus chemotherapy (etoposide-carboplatin); QALY: quality-adjusted life year.

### 3.2 Sensitivity analysis

#### 3.2.1 One-way sensitivity analysis

**FIGURE 2:**
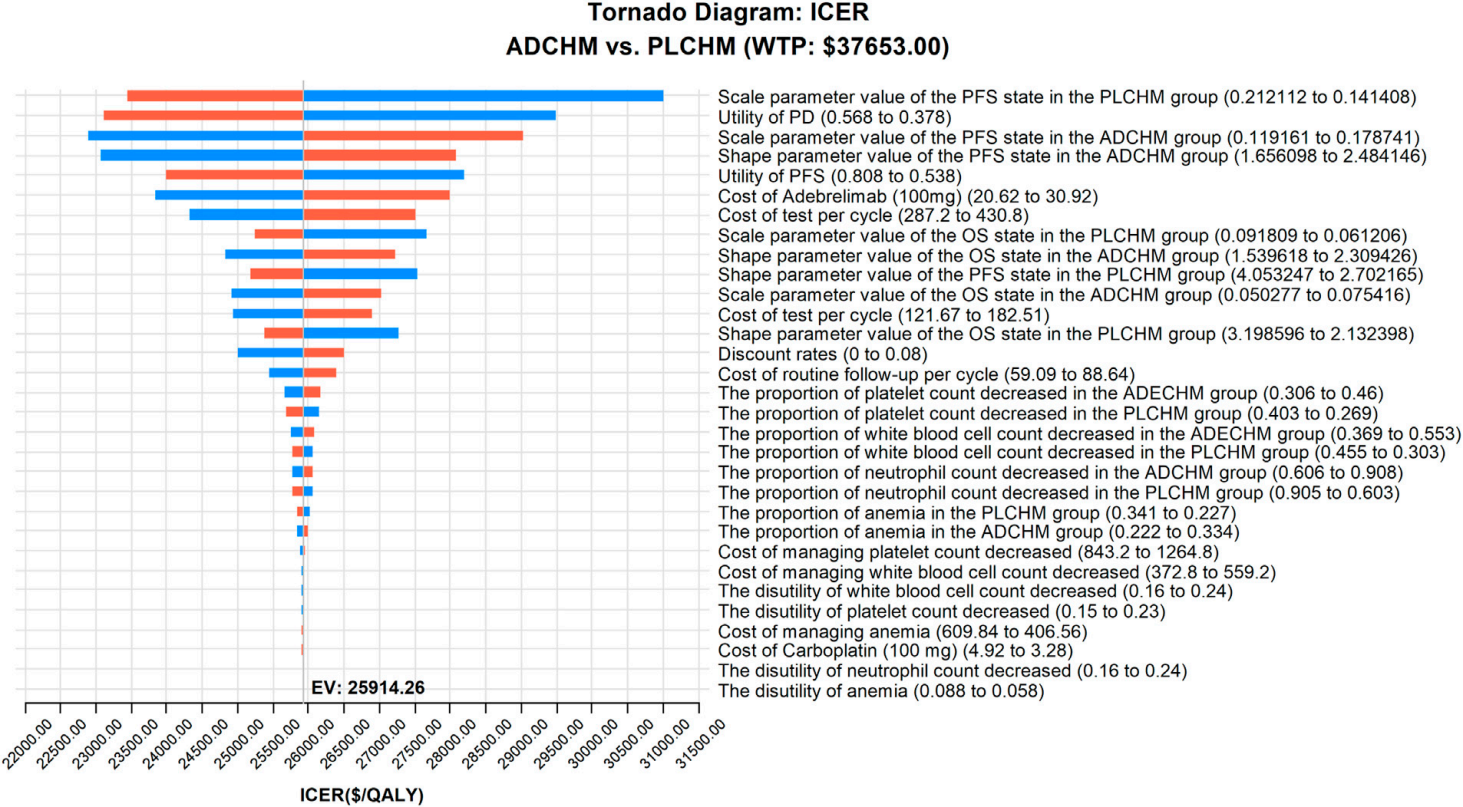
One-way sensitivity analyses of ADCHM in comparison with PLCHM. ADCHM: adebrelimab in combination with chemotherapy (etoposide-carboplatin); ES-SCLC: extensive-stage small-cell lung cancer; ICER: incremental cost-effectiveness ratio; PD: progressive disease; PFS: Progression-free survival; PLCHM: placebo plus chemotherapy (etoposide-carboplatin).

The outcomes of the one-way sensitivity analysis based on the model are presented in the tornado diagram (Figure 2), and the most influential variables were the scale parameter value of the PFS state in the PLCHM group, the utility value of PD, and the scale parameter value of the PFS state in the ADCHM group. Despite changing the values of these parameters, the ICER remained consistently below our predetermined WTP threshold. The variables exerting a relatively small impact on the results were the shape parameter value of the PFS state in the ADCHM group, the utility value of PFS, and the price of adebrelimab (100 mg).

#### 3.2.2 PSA

**FIGURE 3:**
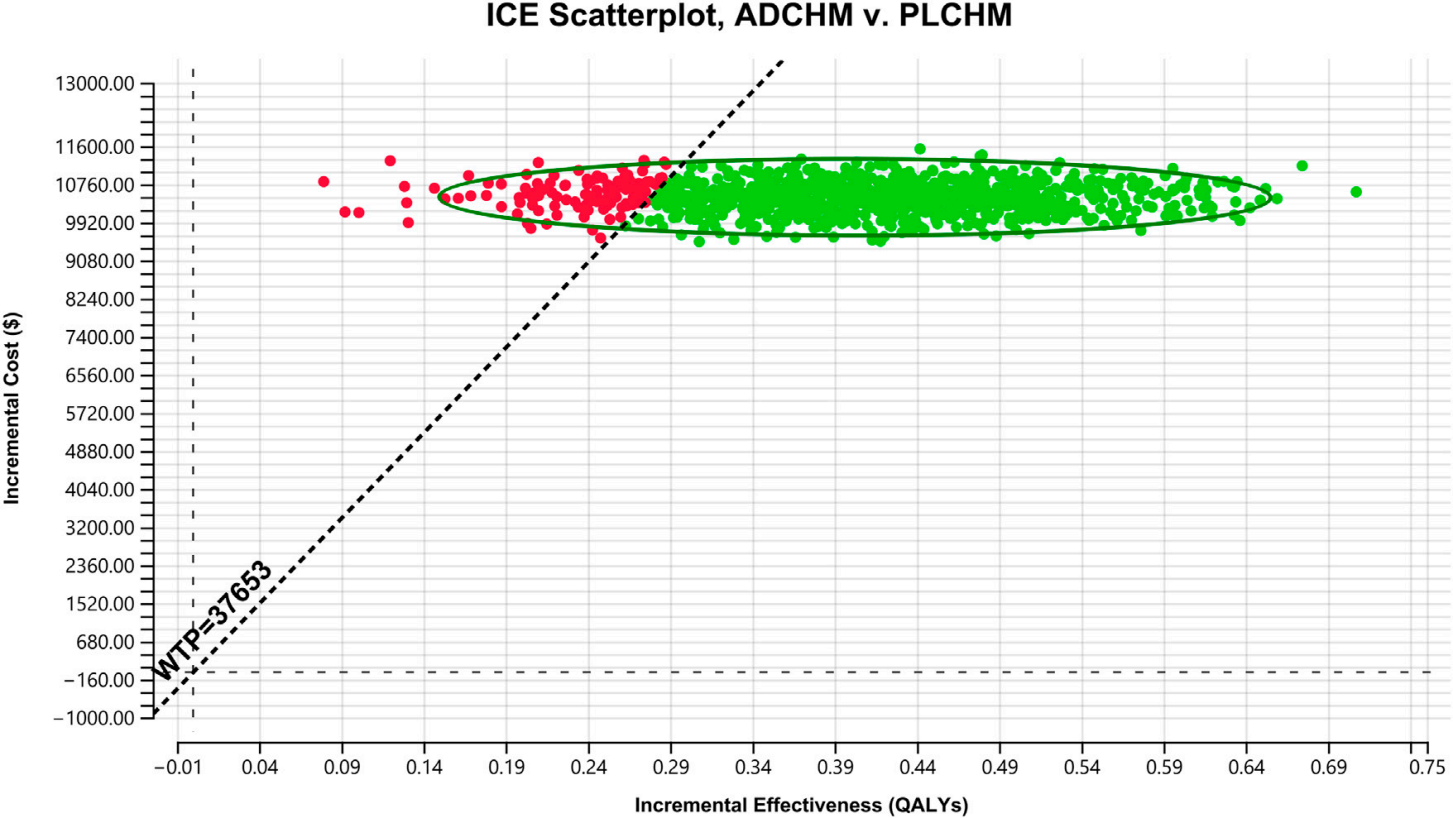
A probabilistic scatter plot of the ICER between the ADCHM group and the PLCHM group. Each point means the ICER for 1 simulation. Ellipses are used to indicate 95% confidence intervals. Points that lie below the ICER threshold represent cost-effective simulations. ADCHM: adebrelimab in combination with chemotherapy (etoposide-carboplatin); PLCHM: placebo plus chemotherapy (etoposide-carboplatin); QALYs, quality-adjusted life years; ICER: incremental cost-effectiveness ratio; WTP: willingness-to-pay.

**FIGURE 4:**
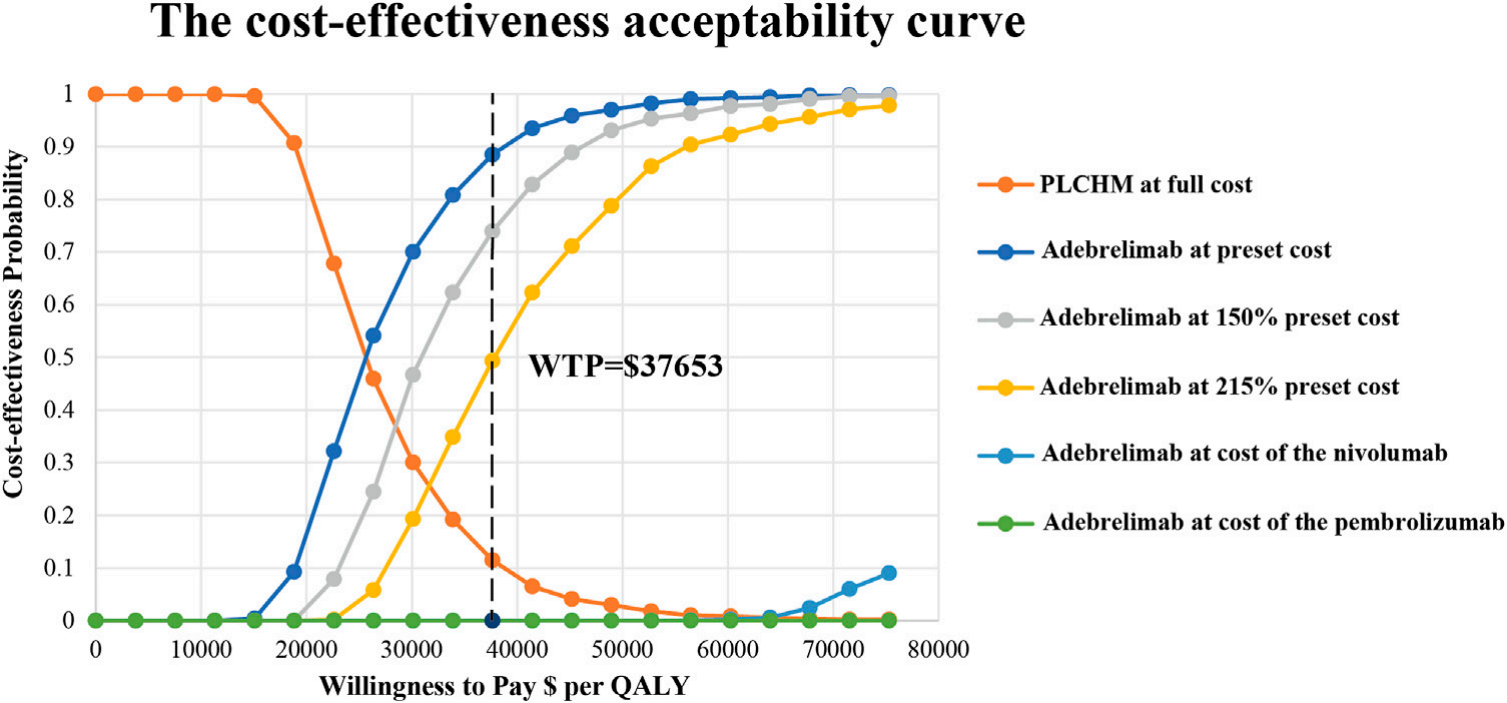
The cost-effectiveness acceptability curves for the ADCHM treatment option compared with the PLCHM treatment option. ADCHM: adebrelimab in combination with chemotherapy (etoposide-carboplatin); PLCHM: placebo plus chemotherapy (etoposide-carboplatin); QALY, quality-adjusted life year; WTP: willingness-to-pay.

The results of the PSA are presented in the scatter plot (Figure 3) and the cost-effectiveness acceptance curve (Figure 4). The probability that the ADCHM group was cost-effective as compared to the PLCHM group when the WTP threshold was $37,653/QALY was 89.1%. The probability of cost-effectiveness of ADCHM was 74.1% when the cost of adebrelimab (100 mg) was set at 1.5 times its original price. When adebrelimab (100 mg) was priced at $55.40, 2.15 times its original price, the probability that ADCHM treatment for ES-SCLC remained cost-effective as compared to PLCHM was 50%. When the market price of pembrolizumab or nivolumab was used as the reference for adebrelimab, the probability of ADCHM’s cost-effectiveness relative to PLCHM was 0.

### 3.3 Subgroup analyses

For most subgroups, the ICER for the ADCHM group as compared to the PLCHM group was below the WTP threshold of $37,653/QALY, ranging from $21,697/QALY in patients who never smoked (probability of cost-effectiveness, 93.7%) to $33099/QALY for PD-L1 tumor proportion score ≥1% (probability of cost-effectiveness, 63.1%). Only in the subgroup of patients with liver metastases, the ICER of the ADCHM group as compared to that of the PLCHM group was higher than $37,653/QALY, reaching $41,617/QALY (probability of cost-effectiveness, 46.3%) (Table 2).

## 4 Discussion

According to the guidelines for the management of primary lung cancer (The General Office of the National Health and Health Commission, 2022), chemotherapy (etoposide combined with carboplatin or cisplatin) in combination with a PD-L1 inhibitor is recommended as the first-line treatment for patients with ES-SCLC. To our knowledge, only three studies have evaluated the cost-effectiveness of combination chemotherapy with PD-L1 inhibitors as a first-line regimen for ES-SCLC from the perspective of the Chinese health system (Li et al., 2019; Liu and Kang, 2022; Tong et al., 2022). However, their results suggest that PD-L1 inhibitor combination chemotherapy is unlikely to be cost-effective for ES-SCLC. Several studies (Zhou et al., 2019; Zhang L et al., 2020; Liu et al.,2021a; Ding et al., 2021; Lin et al., 2021; Wang et al., 2021; Zhu et al., 2021) evaluated the economics of PD-L1 inhibitor from a perspective outside of China showing that PD-L1 inhibitor combined with chemotherapy as the first-line regimen for ES-SCLC is not cost-effective as compared to chemotherapy, whereby the price of the PD-L1 inhibitor has a significant impact on the outcomes of the model, consistent with the findings in China.

A key factor that makes PD-L1 inhibitor plus chemotherapy a cost-effective option for treating ES-SCLC as compared to chemotherapy alone is the price of PD-L1 inhibitors in China. We inferred that the price of adebrelimab confers a great advantage over other PD-L1 inhibitors imported from abroad as it is an indigenously-developed PD-L1 inhibitor in China. In the CAPSTONE-1 trial (Wang et al., 2022), Wang et al. used adebrelimab for the first time as a first-line treatment option for patients with ES-SCLC, and their results suggested that ADCHM as compared to PLCHM as a first-line treatment option significantly improved the OS of previously untreated ES-SCLC patients. The median OS was significantly longer in the ADCHM group relative to the PLCHM group (15.3 months vs. 12.8 months, respectively); OS was higher in the ADCHM group than in the PLCHM group at both 12 and 24 months. The ADCHM group showed a reduced risk of progression or death, a higher objective remission rate, and a longer duration of remission. The safety of the combination of adebrelimab and chemotherapy was manageable, with a low incidence of treatment discontinuation due to adverse events. Thus, adebrelimab, a PD-L1 inhibitor, is a potential therapeutic option for ES-SCLC. However, the high prices of PD-L1 inhibitors (including adebrelimab) have significantly increased healthcare costs, thereby making them an uneconomical treatment option, especially in countries with limited healthcare resources, such as China. Therefore, it is essential to evaluate the cost-effectiveness of ADCHM for ES-SCLC. Based on the results of the CAPSTONE-1 trial (Wang et al., 2022), our findings suggested that ADCHM is a cost-effective first-line treatment option for ES-SCLC as compared to PLCHM. The results of the subgroup analysis showed that most subgroups of patients preferred treatment with ADCHM owing to >50% probability of cost-effectiveness as compared to PLCHM, except for subgroups with liver metastases. This is beneficial for patients with ES-SCLC, as it is the first cost-effective treatment option with PD-L1 inhibitors, a major innovative point highlighted in our study.

We were unable to obtain the price of adebrelimab because it is not yet on the market. The price of adebrelimab in our model was assumed based on the price of other indigenously developed PD-L1 inhibitors in China. Therefore, we varied the price of adebrelimab to obtain different results for the cost-effectiveness of ADCHM for treating ES-SCLC. The different cost-effectiveness results obtained from the different price settings for adebrelimab are expected to provide an important reference for the Chinese health insurance authorities when negotiating the price of adebrelimab. The results of the probabilistic sensitivity analysis showed that ADCHM would no longer remain a cost-effective treatment option if the price of adebrelimab (100 mg) goes beyond $54.40.

The selection of comparators in the model is an important issue to consider when performing a cost-effectiveness analysis. The combination of durvalumab or atezolizumab with chemotherapy as a first-line treatment option for ES-SCLC has been approved by the Food and Drug Administration but this was not assessed in our study (Oronsky et al., 2022). From a Chinese perspective analysis, neither treatment option is cost-effective compared to chemotherapy (Li et al., 2019; Liu and Kang, 2022; Tong et al., 2022). Liu et al. (Liu and Kang, 2022) concluded that durvalumab would require a 90% price reduction to remain cost-effective in the presence of the patient assistance program, while a larger price reduction would be required in the absence of the assistance program. Similarly, Li et al. (2019) concluded that atezolizumab would require a price reduction of 80% or more to become cost-effective. They did not consider the important context of the medical insurance reimbursement, which is consistent with our understanding from the Fujian Provincial Medical Insurance Bureau (http://ybj.fujian.gov.cn/) that neither dulvalumab nor atezolizumab is included in the medical insurance reimbursement list. Thus we believe it is reasonable to select chemotherapy as a comparator for the cost-effectiveness analysis of ADCEHM.

Our study has some limitations. First, owing to the lack of long-term survival data, we used a log-logistic survival model to infer survival tails beyond the observed time horizon, which may not accurately reflect real-world settings. Our cost-effectiveness analysis will be updated when long-term survival data are reported. Second, when patients experience disease progression, we placed some of them on second-line chemotherapy and others on best supportive care due to the lack of relevant survival data for the enrolled patients. Additionally, the duration of second-line chemotherapy was based on the findings of Zhao et al. (2019). This may not accurately reflect the current clinical practice conditions. We will analyze this issue further when relevant treatment costs and survival data for patients after progression are available. Third, we only considered adverse reactions of grade 3 or higher with a probability of occurrence greater than 5% in the model. We assumed that low-probability adverse events would not alter our conclusions. Sensitivity analyses also showed that the results of the model were insensitive to the parameters associated with adverse reactions (including incidence, cost of management, and disutility). Fourth, to simplify the model, we assumed a patient weight of 65 kg, a body surface area of 1.72 m^2^, and a creatinine clearance rate of 70 ml/min; one-way sensitivity analyses showed that the model results were insensitive to these parameters. Fifth, patients were allowed to undergo prophylactic cranial irradiation during the maintenance phase of treatment but prophylactic cranial irradiation was not included in this model owing to the small number of patients receiving brain irradiation in the CAPSTONE-1 trial and the lack of relevant treatment data. Sixth and the biggest limitation of the model is the lack of the actual price of adebrelimab since it is not yet available; we shall update our analysis when the price of adebrelimab is available. Seventh, we did not consider the direct transition from the PFS state to the death state in the Markov model, which may have an inevitable effect on the results of our model. Finally, we assumed that the survival function was consistent for all subgroups in the PLCHM group and constructed the survival function for each subgroup in the ADCHM group from subgroup-specific hazard ratios, which differed from the true survival function, owing to the lack of Kaplan-Meier survival curves for all subgroups in the CAPSTONE-1 trial. This subgroup analysis is an exploratory study, and thus, the results should be interpreted with caution. Despite these limitations, our findings provide a valuable reference for Chinese policymakers for formulating first-line treatment for advanced or metastatic ES-SCLC.

## 5 Conclusion

At present, ADCHM is not recommended as a first-line treatment option in the relevant ES-SCLC guidelines, as it is still under institutional evaluation. However, from the perspective of the Chinese healthcare system, our findings suggest that ADCHM is a cost-effective treatment option as the first-line treatment for ES-SCLC as compared to conventional chemotherapy. Our results provide an important economic rationale for the Chinese healthcare system to consider ADCHM as the first-line treatment option for ES-SCLC and the post-marketing pricing of adebrelimab.

## Data Availability

The original contributions presented in the study are included in the article/Supplementary Material, further inquiries can be directed to the corresponding authors.
