# Supplementary material for: Cost-effectiveness analysis of adebrelimab combined with chemotherapy for extensive-stage small cell lung cancer
Source: Front Pharmacol. 2022 Oct 26;13:1019826. doi: 10.3389/fphar.2022.1019826 (PMC9643856; doi:10.3389/fphar.2022.1019826)
Supplement: Supplementary file 1 [file Table1.pdf]

Supplementary Material

Cost-Effectiveness Analysis of Adebrelimab Combined with Chemotherapy for Extensive-stage Small Cell Lung Cancer

1.Supplementary Table SA. CHEERS 2022 Checklist.
2.Supplementary Table SB. Comparison of survival models.
3.Supplementary Figure SA. Results of the survival curve fit the ADCHM group, PLCHM group.
4.Supplementary Figure SB. The baseline characteristics of patients enrolled in the CAPSTONE-1 trial.

1.Supplementary Table SA. CHEERS 2022 Checklist.

Topic	No.	Item	Location where item is reported
**Title**			
	1	Identify the study as an economic evaluation and specify the interventions being compared.	Page1 Line1-2
**Abstract**			
	2	Provide a structured summary that highlights context, key methods, results, and alternative analyses.	Page2 Line42-68
**Introduction**			
**Background and objectives**	3	Give the context for the study, the study question, and its practical relevance for decision making in policy or practice.	Page3 Line86-123
**Methods**			
**Health economic analysis plan**	4	Indicate whether a health economic analysis plan was developed and where available.	Not applicable
**Study population**	5	Describe characteristics of the study population (such as age range, demographics, socioeconomic, or clinical characteristics).	Page4 Line168-174
**Setting and location**	6	Provide relevant contextual information that may influence findings.	Page4-5 Line 168-169
**Comparators**	7	Describe the interventions or strategies being compared and why chosen.	Page4-5 Line174-180
**Perspective**	8	State the perspective(s) adopted by the study and why chosen.	Page4 Line159-160
**Time horizon**	9	State the time horizon for the study and why appropriate.	Page4 Line152-153
**Discount rate**	10	Report the discount rate(s) and reason chosen.	Page5 Line214-215
**Selection of outcomes**	11	Describe what outcomes were used as the measure(s) of benefit(s) and harm(s).	Page4 Line157-159
**Measurement of outcomes**	12	Describe how outcomes used to capture benefit(s) and harm(s) were measured.	Page4 Line160-164
**Valuation of outcomes**	13	Describe the population and methods used to measure and value outcomes.	Page5 Line207-213
**Measurement and valuation of resources and costs**	14	Describe how costs were valued.	Page5 Line197-205

Topic	No.	Item	Location where item is reported
**Currency, price date, and conversion**	15	Report the dates of the estimated resource quantities and unit costs, plus the currency and year of conversion.	Page5 Line205-207
**Rationale and description of model**	16	If modelling is used, describe in detail and why used. Report if the model is publicly available and where it can be accessed.	Page4 Line148-164
**Analytics and assumptions**	17	Describe any methods for analysing or statistically transforming data, any extrapolation methods, and approaches for validating any model used.	Page3-4 Line129-147
**Characterising heterogeneity**	18	Describe any methods used for estimating how the results of the study vary for subgroups.	Page6 Line245-259
**Characterising distributional effects**	19	Describe how impacts are distributed across different individuals or adjustments made to reflect priority populations.	Page6 Line219-226
**Characterising uncertainty**	20	Describe methods to characterise any sources of uncertainty in the analysis.	Page6 Line227-243
**Approach to engagement with patients and others affected by the study**	21	Describe any approaches to engage patients or service recipients, the general public, communities, or stakeholders (such as clinicians or payers) in the design of the study.	Not applicable
**Results**			
**Study parameters**	22	Report all analytic inputs (such as values, ranges, references) including uncertainty or distributional assumptions.	Table 1
**Summary of main results**	23	Report the mean values for the main categories of costs and outcomes of interest and summarise them in the most appropriate overall measure.	Page6-7 Line261-270, Table 2
**Effect of uncertainty**	24	Describe how uncertainty about analytic judgments, inputs, or projections affect findings. Report the effect of choice of discount rate and time horizon, if applicable.	Page7 Line 272-297
**Effect of engagement with patients and others affected by the study**	25	Report on any difference patient/service recipient, general public, community, or stakeholder involvement made to the approach or findings of the study	Not applicable
**Discussion**			

Topic	No.	Item	Location where item is reported
**Study findings, limitations, generalisability, and current knowledge**	26	Report key findings, limitations, ethical or equity considerations not captured, and how these could affect patients, policy, or practice.	Page10-11 Line419-444
**Other relevant information**			
**Source of funding**	27	Describe how the study was funded and any role of the funder in the identification, design, conduct, and reporting of the analysis	Page11 Line462-466
**Conflicts of interest**	28	Report authors conflicts of interest according to journal or International Committee of Medical Journal Editors requirements.	Page11 Line467-468

2.Supplementary Table SB. Comparison of survival models.

	AIC		BIC	
	ADCHM	PLCHM	ADCHM	PLCHM
OS				
Exponential	1239.562	1429.548	1243.019	1433.015
Weibull	1216.739	1351.165	1223.837	1358.167
Log-normal	1207.351	1366.189	1214.176	1373.046
Log-logistic	1207.036	1344.776	1214.147	1351.429
PFS				
Exponential	1167.226	1136.5027	1170.684	1139.970
Weibull	1161.150	1054.8204	1168.131	1061.813
Log-normal	1111.201	1020.6831	1118.029	1027.542
Log-logistic	1104.136	994.0828	1110.695	1000.699

AIC: Akaike information criterion; ADCHM: adebrelimab in combination with chemotherapy (etoposide-carboplatin); BIC: Bayesian Information Criterion; OS: Overall survival;
PFS: Progression-free survival; PLCHM: placebo plus chemotherapy (etoposide-carboplatin).

3.Supplementary Figure SA. Results of the survival curve fit the ADCHM group, PLCHM group.

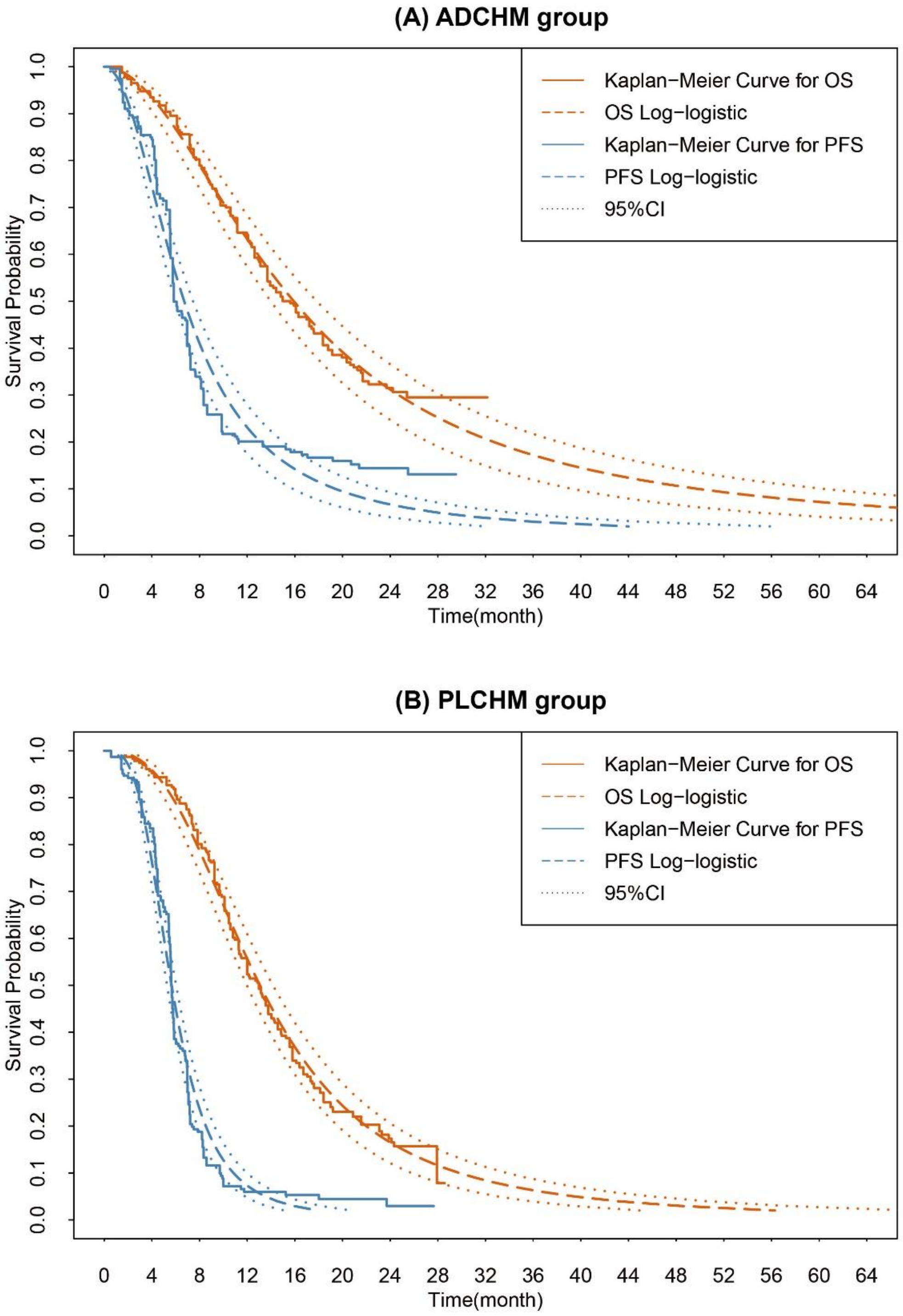

95%CI: 95% confidence interval; ADCHM: adebrelimab in combination with chemotherapy (etoposide-carboplatin); OS: Overall survival; PFS: Progression-free survival; PLCHM: placebo plus chemotherapy (etoposide-carboplatin).

4.Supplementary Figure SB. The baseline characteristics of patients enrolled in the CAPSTONE-1 trial.

	Adebrelimab group (n=230)	Placebo group (n=232)
**Age, years**		
Median (IQR)	62 (55 – 66)	62 (56 – 67)
<65	155 (67%)	147 (63%)
⩾65	75 (33%)	85 (37%)
**Sex**		
Male	184 (80%)	188 (81%)
Female	46 (20%)	44 (19%)
**ECOG performance status**		
0	33 (14%)	30 (13%)
1	197 (86%)	202 (87%)
**Smoking history**		
Never smoked	50 (22%)	53 (23%)
Former smoker	180 (78%)	178 (77%)
Current smoker	0	1 (<1%)
**Disease stage**		
III	8 (3%)	6 (3%)
IV	222 (97%)	226 (97%)
**Lactate dehydrogenase at enrolment**		
⩽ULN	116 (50%)	115 (50%)
>ULN	114 (50%)	117 (50%)
**Brain metastases**		
Yes*	5 (2%)	5 (2%)
No	225 (98%)	227 (98%)
**Liver metastases**		
Yes	73 (32%)	74 (32%)
No	157 (68%)	158 (68%)
**PD-L1 tumour proportion score**		
<1%	196 (85%)	200 (86%)
⩾1%	24 (10%)	20 (9%)
Not evaluable	10 (4%)	12 (5%)

Data are n (%) or median (IQR). ECOG=Eastern Cooperative Oncology Group. ULN=upper normal limit. *In each group, three (60%) of five patients with brain metastases received radiotherapy and two (40%) received surgery at diagnosis.

PD-L1, programmed cell death receptor ligand-1.
